# The contribution of E3D imaging integrated with robotic navigation: analysis of the first 80 consecutive posterior spinal fusion cases

**DOI:** 10.1007/s11701-024-02014-5

**Published:** 2024-07-08

**Authors:** Jeffrey J. Stewart, Paul Asdourian, Bradley Moatz, Mosope Soda, Mesfin Lemma, Bryan W. Cunningham, Daina M. Brooks, Paul C. McAfee

**Affiliations:** 1 Department of Orthopaedic Surgery, MedStar Union Memorial Hospital, 3333 North Calvert Street, Suite 400, Baltimore, MD 21218 USA; 2 Department of Neurosurgery, Georgetown School of Medicine, Washington, DC USA

**Keywords:** Robotic navigation, Spinal deformity, Posterior spinal fusion, Intraoperative CT registration

## Abstract

Eighty consecutive complex spinal robotic cases utilizing intraoperative 3D CT imaging (E3D, Group 2) were compared to 80 age-matched controls using the Excelsius robot alone with C-arm Fluoroscopic registration (Robot Only, Group 1). The demographics between the two groups were similar—severity of deformity, ASA Score for general anesthesia, patient age, gender, number of spinal levels instrumented, number of patients with prior spinal surgery, and amount of neurologic compression. The intraoperative CT scanning added several objective factors improving patient safety. There were significantly fewer complications in the E3D group with only 3 of 80 (4%) patients requiring a return to the operating room compared to 11 of 80 (14%) patients in the Robot Only Group requiring repeat surgery for implant related problems (Chi squared analysis = 5.00, *p* = 0.025). There was a significant reduction the amount of fluoroscopy time in the E3D Group (36 s, range 4–102 s) compared to Robot only group (51 s, range 15–160 s) (*p* = 0.0001). There was also shorter mean operative time in the E3D group (257 ± 59.5 min) compared to the robot only group (306 ± 73.8 min) due to much faster registration time (45 s). A longer registration time was required in the Robot only group to register each vertebral level with AP and Lateral fluoroscopy shots. The estimated blood loss was also significantly lower in Group 2 (mean 345 ± 225 ml) vs Group 1 (474 ± 397 ml) (*p* = 0.012). The mean hospital length of stay was also significantly shorter for Group 2 (3.77 ± 1.86 days) compared to Group 1 (5.16 ± 3.40) (*p* = 0.022). There was no significant difference in the number of interbody implants nor corrective osteotomies in both groups—Robot only 52 cases vs. 42 cases in E3D group.

*Level of evidence*: IV, Retrospective review.

## Introduction

The Excelsius (E3D) intraoperative imaging was introduced as a three in one platform to combine intraoperative CT registration with digital radiography and fluoroscopy integrated with robotic navigation. Rather than list the specific theoretical parameters that allow application of this innovative technology we reviewed the actual practical clinical contributions of E3D in our first 80 consecutive cases. Our particular academic practice with several residency training programs has a heavy case mix with a large number of deformity cases (average over 7 vertebral levels). We reported our first 100 deformity robotic cases previously [1]—31 revision cases with prior instrumentation in place, 28 cases with S2AI pelvic fixation, 1043 consecutive thoracolumbar pedicle screws and 53 S2AI screws with zero reoperations required due to malpositioning of screws.

## Materials and methods

Eighty consecutive complex spinal robotic cases utilizing intraoperative 3D CT imaging (E3D, Group 2) were compared to 80 age -matched controls using Excelsius GPS robot alone (Group 1). The demographics between the two groups were similar—severity of deformity, ASA Stage, patient age, gender, number of spinal levels instrumented, and amount of neurologic compression.

This series of 80 cases highlights the advantages when the E3D imaging system ls used to register and help create the virtual Excelsius construct for spinal navigation and robotics. The E3D and integrated Excelsius robot are a platform and no doubt additional advantages will be seen in the future. The first 80 cases of E3D in deformity correction were analyzed in detail by quantifying the data and comparing them to a series of C-arm registered 80 deformity cases in the same institution with the same four surgeons. The data include the learning curves of four surgeons adopting the intraoperative 3D CT imaging integrated with the Excelsius spinal robot.

### Criteria for device-related complication

In order to objectively identify device related complications we utilized the FDA criteria for device related problems—no implant revisions, no removals, any cases requiring repeat surgery for implant loosening, infection, or cases requiring additional fusion or stability supplementation. Finally, any cases that required repeat surgery in order to perform a more complete decompression due to nerve root irritation or exacerbation of stenotic symptoms after the initial surgical deformity reduction. We utilized the FDA criteria for subsequent surgical interventions related to the robotically guided or navigated spinal implants [2].

The most important advantage is that the work flow with E3D allows for the CT to be performed AFTER the patient is in the exact position for spinal instrumentation. The patient is prone with the exact amount of desired thoracic kyphosis and lumbar lordosis, and the soft tissue dissection is already completed. Because the entire imaging could be completed in 45 s, the respirations can be held until the virtual image is completed and transferred to the robotic computer. Contrast this with the workflow without the E3D—a CT scan is performed an average of 3 days before surgery, the patient is ambulatory or transferred to the OR with possibly an unstable spine, and there is 3 cm or more excursion of the chest cage, ribs, thoracic pedicles on inspiration. Using intraoperative fluoroscopy without E3D, even in the hands of an experienced X-ray technologist registration can require as much as 45 min—as perfect AP and Lateral C-arm images are required for each vertebra from T4 to L4, for example.

**Fig. 1:**
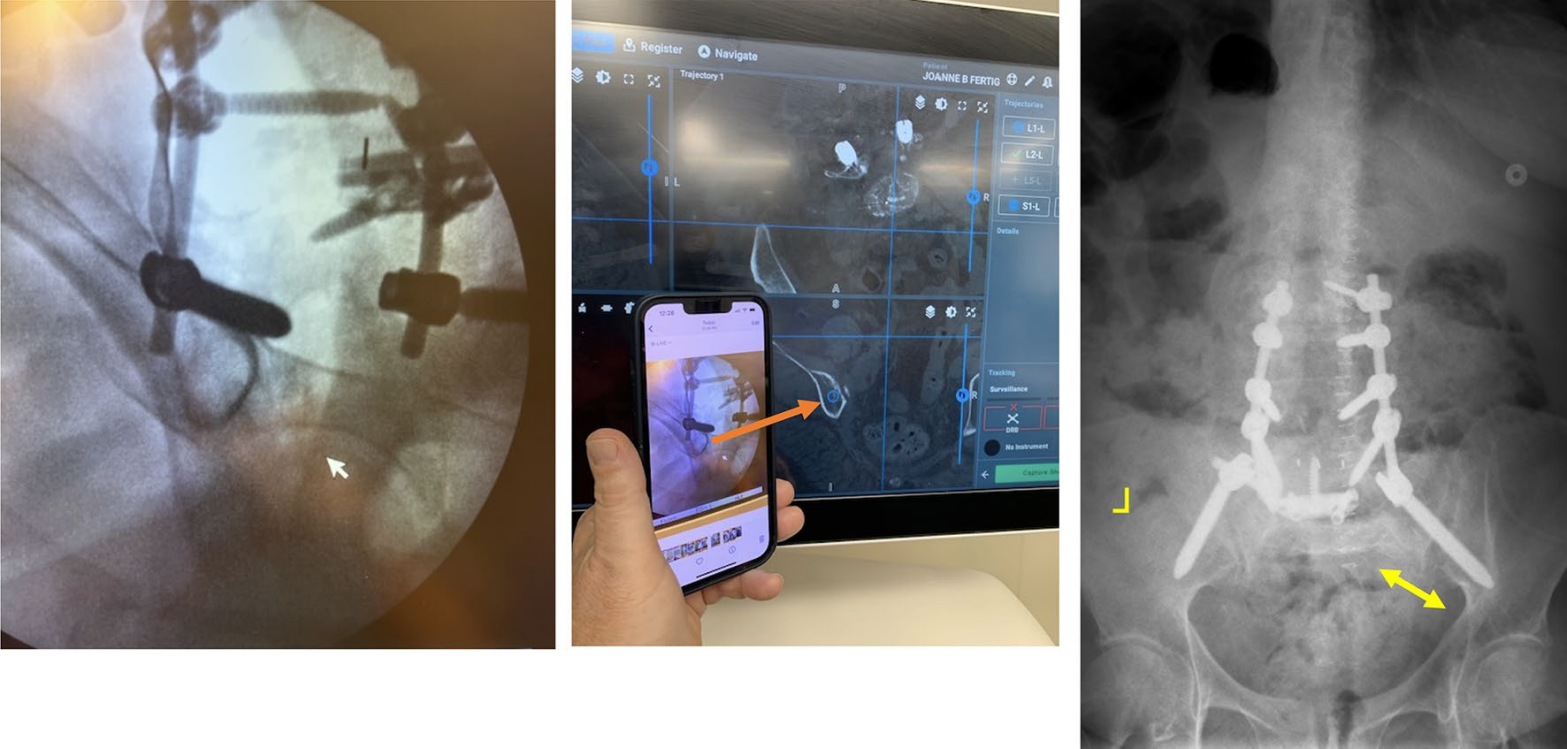
The preoperative CT imaging for pedicle and sacral alar iliac screw positioning was acquired in the supine position (Group 1 patient). This became the template for the virtual architecture of the pelvis for S2AI screw placement. Following induction with general anesthesia, the patient was placed in the prone position on the operating table with intraoperative CT and automatic registration. Unfortunately, due to unsuspected SI joint ligamentous insufficiency, the pelvis opened up like a book. This precipitated the operative navigation system to miscalculate the trajectory of the left S2AI screw which was placed medial to the inner table. Therefore, the right SI joint was unstable and opened spinopelvic dissociation, skewing the navigation. Fortunately, this medial pathway was identified and corrected at surgery. The registration of the navigation system was repeated in the prone position. After correction the tip of the screw can be seen at the pelvic teardrop. Unsuspected separation of right sacroiliac joint occurred at the start of surgery when the patient was turned into the prone position under anesthesia. The virtual spine reconstruction on the computer was inaccurate as it was based on a preoperative CT performed 2 days earlier as an outpatient. Group 1 patient. The misplacement of the left iliac screw would have been avoided if the registration and computer rendering had been performed with E3D

We have had fracture patients with unsuspected sacroiliac ligament disruption actually have the pelvis “open up like a book” when placed prone on the operating table—this renders the preoperative ambulatory CT inaccurate whereas an intraoperative CT from the E3D provides perfect anatomic orientation (Fig. 1).

## Results

The demographics between the two groups were similar (Table 1)—severity of deformity, ASA Stage, patient age, gender, number of spinal levels instrumented, and amount of neurologic compression.

**Table 1:** The demographics between the two groups of 80 patients each demonstrate that the two populations of deformity spine patients undergoing robotic surgery were remarkably similar

	Robot	E3D	*p* value
Patients	80	80	
Age			
Mean	65.1 ± 12.4	63.9 ± 12.5	0.612
Range	18–86	19–83	
Female	42 (52.5%)	35 (44.9%)	
Male	38 (47.5%)	43 (55.1%)	
BMI	31.16 ± 6.48	29.59 ± 6.13	0.320
ASA classification	2.75 ± 0.52	2.56 ± 0.55	1.000
HTN	56 (70.0%)	52 (66.7%)	0.652
*Same 4 surgeons*			
CAD	15 (18.8%)	15 (19.2%)	0.938
CHF	6 (7.5%)	2 (2.6%)	0.157
DVT/PE	11 (13.8%)	4 (5.1%)	0.065
Asthma	16 (20.0%)	10 (12.8%)	0.375
COPD	12 (15.0%)	5 (6.4%)	0.257
OSA	26 (32.5%)	19 (24.4%)	0.257
CVA/TIA	6 (7.5%)	4 (5.1%)	0.540
CKD	4 (5.0%)	3 (3.8%)	0.725
Type II DM	20 (25.0%)	26 (33.3%)	0.249
GERD	42 (52.5%)	36 (46.2%)	0.425
Tobacco use	27 (33.8%)	28 (35.9%)	0.777
*Interbodies/osteotomies*	**52 (0.65** **±** **−0.73)**	**45 (0.58** **±** **−0.63)**	**1.000**
Osteoporosis	2 (2.5%)	1 (1.3%)	0.575

p < 1.000. The number of interbody spacers and osteotomies are nearly equally distributed between Groups 1 and 2 (in Bold). The number of osteotomies and interbody spacers are the most sensitive indication of the magnitude of the surgical reconstruction. This demonstrates that the magnitude of the navigated robotic procedures was approximately equivalent between the two Groups

One of the best measures of the magnitude of the deformity surgery was the number of spinal osteotomies and interbody spacers utilized. No significant difference in the number of interbody implants nor corrective osteotomies in both groups—Robot only 52 cases vs. 42 cases in Integrated Radiographic CT (IRCT) group.

**Fig. 2:**
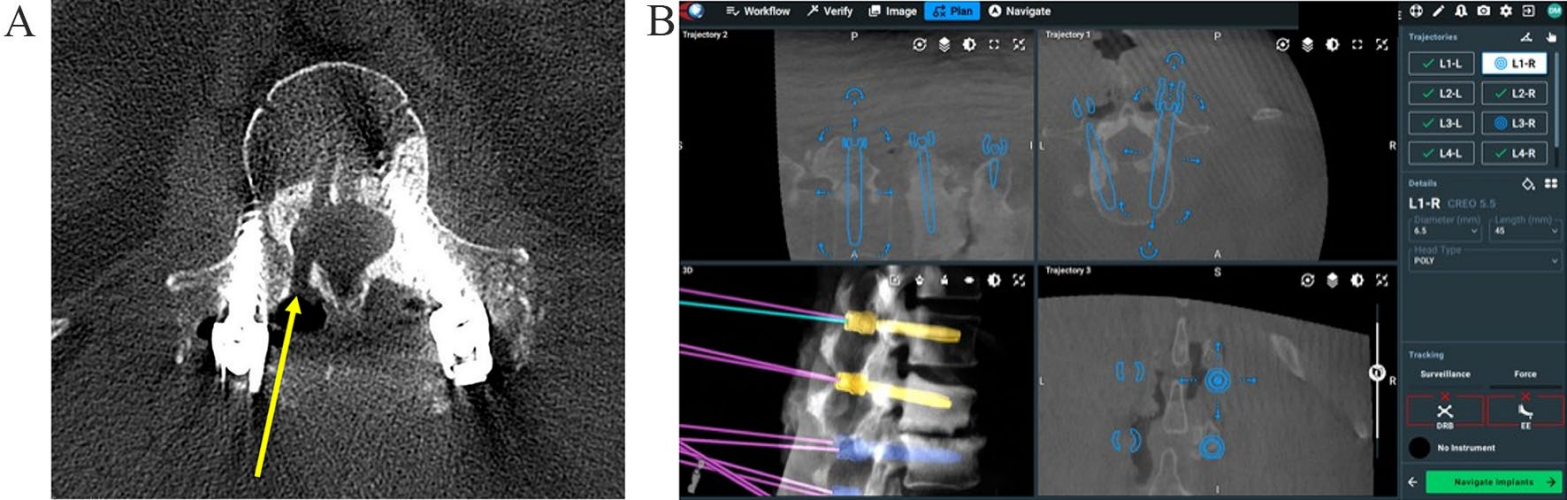
**A** The pedicle screw fixation in L1 was performed at Elsewhere General using robotics. If the surgeons had Excelsius E3D as part of their armamentarium, the intracanal placement of the right L1 pedicle screw could have been avoided. The illustration shows the trajectory of the misplaced screw. The figure also shows the final position of the left and right L1 pedicle screws. **B** In retrospect, the Excelsius plan and virtual image of the right L1 pedicle screw showed “perfect” placement. This is a real problem anytime there is a discrepancy between reality (demonstrated by the E3D image) and the virtual reconstruction based on preoperative CT combined with intraoperative fluoroscopic registration. There was an indication during surgery of a problem as the spinal cord monitoring electrical screw stimulation showed a low threshold—but the definitive way of performing a screw check is with E3D. If the surgeon has an index of suspicion then an E3D screw check with a shallow guidewire in the outer cortex is preferred to retrospective E3D imaging after the screw is inserted to full depth

Table 2 is a listing of the major comparisons showing objectively the contribution of intraoperative imaging to spinal robotics and navigation. The intraoperative CT scanning added several objective factors improving patient safety (Fig. 2).

**Table 2:** The patients in Group 2, E3D had statistically fewer reoperations than the patients in Group 1, C-arm fluoroscopic registration (*p* = 0.0285)

	Robot only (*n* = 80)	E3D (*n* = 80)	*p* value
*FDA criteria for return to OR*			
Revision			
Removal			
Required posterior supp instr			
Required additional neuro decompression			
Number of interbodies/osteotomies			
Total	52	45	
Mean	0.65 ± 0.73	0.58 ± 0.63	1.0000
Mean fluoro time (s)	**51** **±** **26**	**36** **±** **20**	**0.0001**
Mean length of surgery (min)	**306** **±** **73**	**257** **±** **60**	**0.0001**
Mean EBL (ml’s)	**474** **±** **397**	**345** **±** **225**	**0.0130**
FDA criteria—complications	**11 (13.8%)**	**3 (3.8%)**	**0.0285**
Instrumentation related	8	1	
Infection	2	0	
Other	1	2	
Length of stay	**5.16** **±** **3.40**	**3.77** **±** **1.86**	**0.0236**

p< .05 (in Bold) are all significantly more favorable for Group 2 versus Group 1. Significantly less radiation exposure, shorter mean length of surgery duration, mean estimated blood loss. There was also a lower revision surgery rate using FDA criteria and shorter length of hospital stay

They also demonstrated significantly less radiation exposure, less mean blood loss, and had a shorter operative time. Perhaps the most important finding is the shorter length of hospital stay by more than a day for the E3D patients (*p* = 0.0236)

There were significantly fewer complications in Group 2 with only 3 of 80 patients (4%) requiring a return to the operating room compared to 11 of 80 patients (14%) in Group 1 (Chi squared analysis = 5.00, *p* = 0.025).

There was a significant reduction the amount of fluoroscopy time in Group 2 (36 s, range 4–102 s) compared to Robot only group (51 s, range 15–160 s) (*p* < 0.0001). Group 2 had a shorter mean operative time (257 ± 59.5 min) compared to Group 1 (306 ± 73.8 min). The estimated blood loss was also significantly lower in Group 2 (mean 345 ± 225 ml) vs Group 1 (474 ml ± 397) (*p* = 0.012). The mean hospital length of stay was also significantly shorter for Group 2 (3.77 ± 1.86 days) compared to Group 1 (5.16 ± 3.40) (*p* = 0.022).

## Discussion

Meng et al. [3] reported computer navigation versus fluoroscopic guided navigation for thoracic pedicle screw placement utilizing a different CT system—the ISO-C 3D. The incidence of complications such as screw misplacement had a lower incidence in the computer navigated group—10 events out of 290 versus C-arm fluoroscopy 38 events out of 283 (odds ratio = 0.23, *p* < 0.001). Their meta-analysis of 14 publications showed a lower insertion time per screw for computer navigation group compared to the conventional navigated group. The ISO-C 3D can be thought of as a first generational robotic computer navigated system which often required multiple re-registrations, therefore the operative time, estimated blood loss and other secondary outcomes were not significantly improved.

During surgery it is very common for less experienced roboticists to “gently” expose the pedicle screw’s starting position. Unfortunately by retracting the paraspinal muscles even with seemingly little force, the pedicle screw insertion can be misdirected by as much as a centimeter. While this happens in the real situation, at the same time the robotic navigation screen can indicate a perfect screw placement position. However we have found with using the E3D this can be prevented by percutaneous or stab incisional placement of screws. The screws are placed through the paraspinal muscles instead of retracting the paraspinal muscles and inadvertently moving the vertebral target. Then an intraoperative image can be obtained, essentially rebooting or reconfiguring the virtual spine to the actual spine anatomy in near “real time” (approximately 45 s prior to screw placement).

**Fig. 3:**
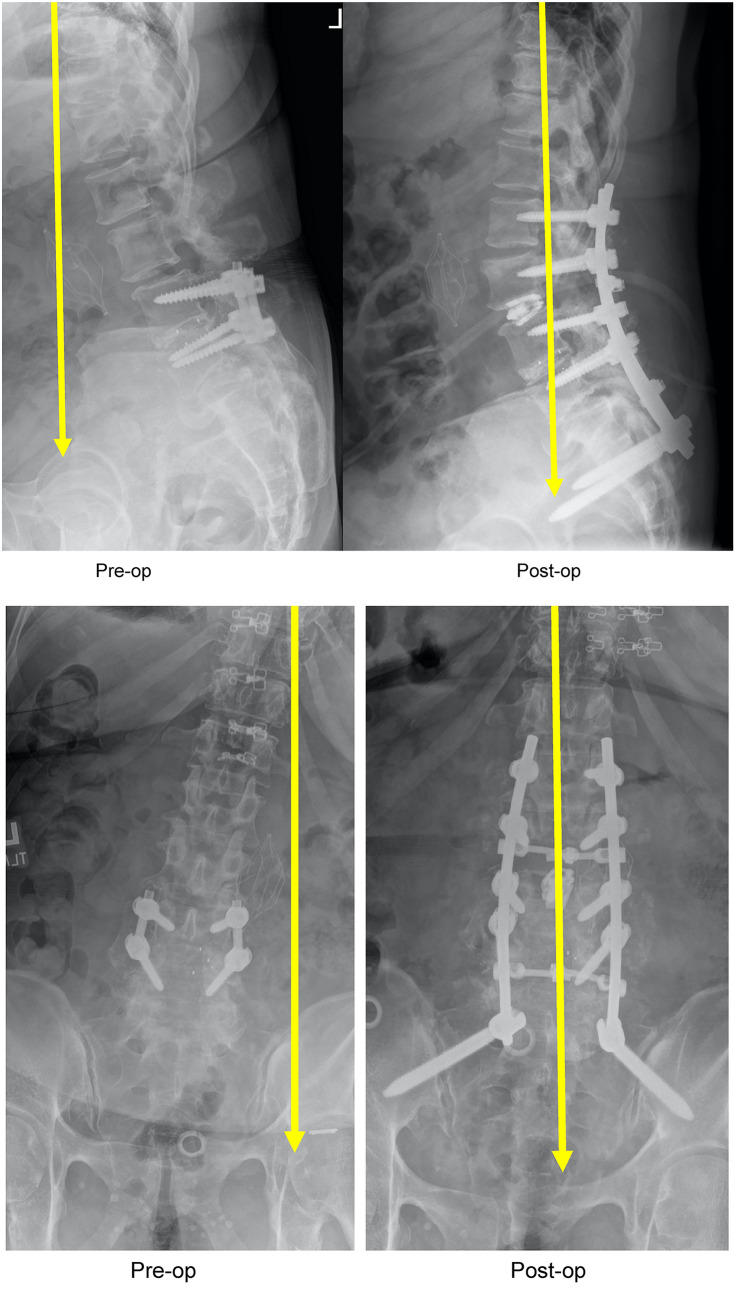
A 67-year-old security guard with neurogenic claudication and difficulty walking underwent an L4–L5 instrumented fusion that caused sagittal and coronal imbalance. Because the treating surgeons from Elsewhere General did not have an intraoperative global image—she was fused with an oblique take off towards the right (Coronal imbalance = 7 cm) and sagittal vertical malalignment of SVL = 8 cm. E3D provided for global visualization and improved assessment of intraoperative realignment. The post-operative standing correction was maintained—perfect sagittal and coronal realignment

**Fig. 4:**
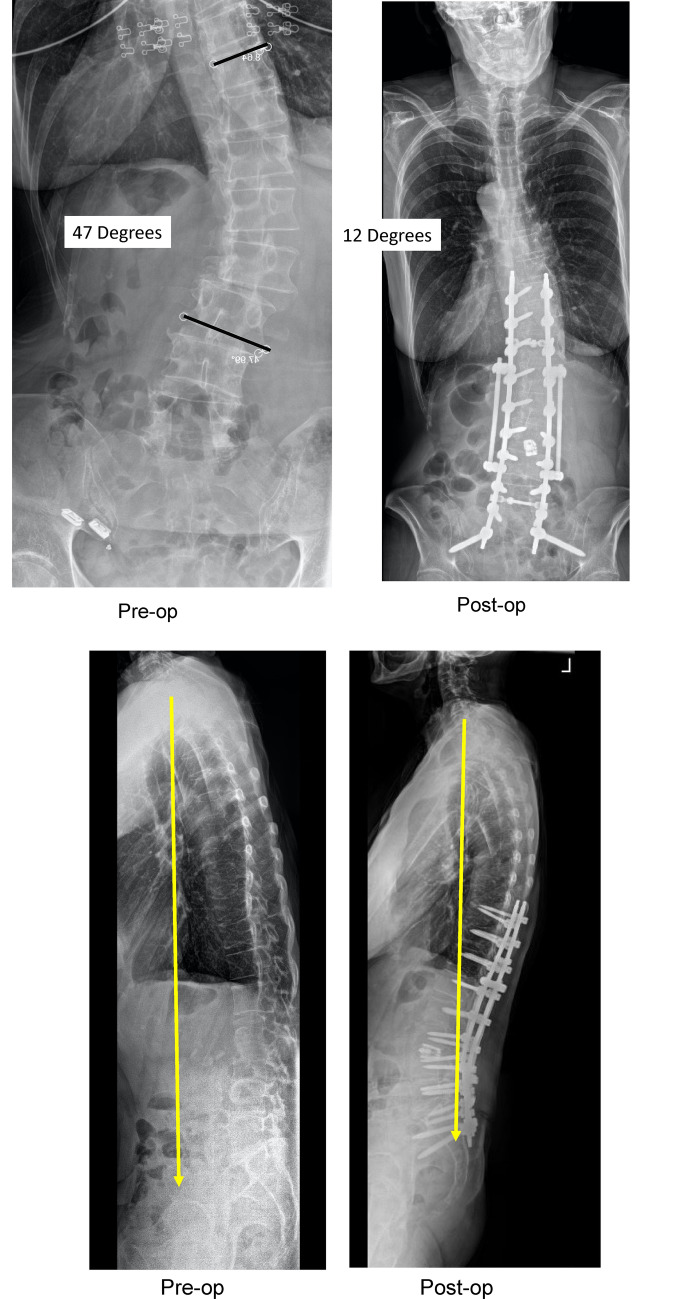
70-year-old woman with scoliosis and flat back syndrome. With E3D global realignment visualization, 236 mm along the spine, and a 50% larger field of view. Successful robotic instrumentation from T10 to the pelvis and L3 spinal osteotomies were performed. In the anteroposterior view her scoliosis was corrected from 47° down to 12°. The lateral view documents global correction of (SVL) sagittal vertical line from 9 cm down to normal. The spinal osteotomy and spinal instrumental shows that her preoperative lumbar kyphosis of 20° was corrected to 46° of lumbar lordosis

The E3D imaging platform is three in one—Cone-Beam CT (CBCT), fluoroscopy, and digital radiography (DR). The application to spinal deformity, T4 to L1 or T10 to pelvis provides a more global visualization for the surgeon, 236 mm along the spine—50% larger field of view compared to legacy systems (Figs. 3, 4).

**Fig. 5:**
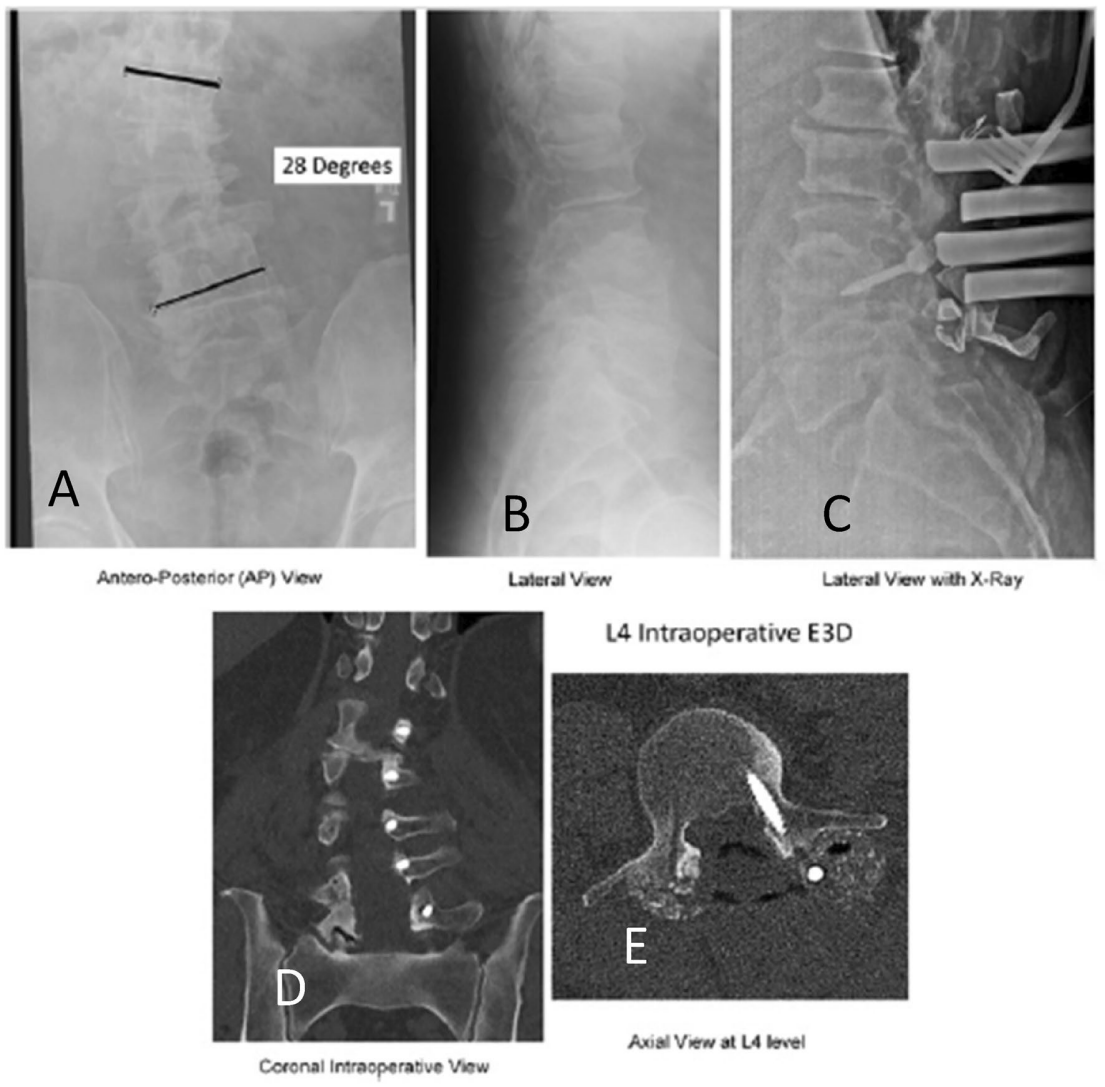
This is a typical AP (**A**) and lateral radiograph (**B**) of a 70 year old man with a 28° scoliosis who underwent posterior decompression, fusion, and instrumentation from L1 to L5. Notice that on the preoperative radiographs that the anatomy is very indistinct and each disk space is narrowed. **C** After placement of the first pedicle screw it was very difficult on AP and Lateral plain radiographs to confirm the correct placement of the first pedicle screw. It is difficult actually to determine even whether the screw is placed in L3 or L4, much less if it is in optimal position. Coronal CT reconstruction (**D**) and axial (**E**) CT images taken intraoperatively with E3D. Notice that there is no doubt that the axial image through L4 demonstrates the correct trajectory of the pedicle screw. In addition the coronal image confirms optimal placement of all 5 pedicle screws on the concave side of the lumbar spine from L1 to L5 before correction with a longitudinal 5.5 mm titanium rod. The E3D imaging leaves no room for ambiguity compared to fluoroscopy and plain digital radiographs, especially in the axial plane

The E3D has two C-shaped gantries, inner and outer, which allow the x-ray beam to rotate a full 360° when used during CBCT. The inner and outer C-shaped gantries can also be nested together to allow the E3D to be position in a lateral position, same as a typical C-arm workflow. This allows the surgeon to fine tune the instrumentation and work around the E3D without moving it in and out away from the operating table (Fig. 5).

The tertiary nature of our deformity cases included 30% revision cases with prior instrumentation—it is critical that the E3D allows for automatic subtraction of the prior metal implants and reduces metal scatter and artifact. We have used other intraoperative imaging systems that provide a good axial image except that the visualization of the spinal canal is totally lost as soon as a pedicle screw is inserted. In a residency training program, this negates the advantage of an intraoperative CT—it is mandatory to be able to confirm/reevaluate screw position relative to the canal. Figure 2 shows a case with an intracanal screw placed by robot at Elsewhere General. Even a resident with limited experience could prevent this complication with E3D, because the visual resolution of the spinal canal is unmistakable.

It is important to mention several limitations of our study. First, it was not possible to separate out either in the operating room record or the radiation records the amount of time utilized during surgery for the spine anatomy registration—this had to be indirectly measured from the total duration of surgery. Second, the two groups overlapped temporally so one might argue that the “learning curve” of robotics more directly influenced the robot only group. However, there were two additional surgeons who adopted robotics primarily in the E3D group, therefore, essentially two surgeons learning curves comprised the Group 1 complications and two additional surgeon’s learning curves were included in the Group 2 experience. Furthermore, the learning curve of adopting the new technology of intraoperative scanning and work flow was only present in the E3D Group. Therefore, this would serve to bias the results against Group 2, the E3D group, which in fact had only three of 80 cases require a return to the operating room for any reason (versus 11 of 80 patients for Group 1).

In our experience with 80 consecutive cases, the more unstable the spine, the more important it is to obtain the virtual construct based on intraoperative position—prone, without paraspinal muscle retraction, without respiratory excursion, after paraspinal dissection is completed, and without dynamic reference base (DRB) movement. In short, the more unstable the spine, the greater the advantage of E3D.

## Data Availability

No datasets were generated or analysed during the current study.
